# A Novel Atoh1 “Self-Terminating” Mouse Model Reveals the Necessity of Proper Atoh1 Level and Duration for Hair Cell Differentiation and Viability

**DOI:** 10.1371/journal.pone.0030358

**Published:** 2012-01-18

**Authors:** Ning Pan, Israt Jahan, Jennifer Kersigo, Jeremy S. Duncan, Benjamin Kopecky, Bernd Fritzsch

**Affiliations:** Department of Biology, University of Iowa, Iowa City, Iowa, United States of America; Universitätsklinikum Carl Gustav Carus an der Technischen Universität Dresden, Germany

## Abstract

Atonal homolog1 (*Atoh1*) is a bHLH transcription factor essential for inner ear hair cell differentiation. Targeted expression of *Atoh1* at various stages in development can result in hair cell differentiation in the ear. However, the level and duration of *Atoh1* expression required for proper hair cell differentiation and maintenance remain unknown. We generated an *Atoh1* conditional knockout (CKO) mouse line using *Tg(Atoh1-cre)*, in which the *cre* expression is driven by an *Atoh1* enhancer element that is regulated by Atoh1 protein to “self-terminate” its expression. The mutant mice show transient, limited expression of *Atoh1* in all hair cells in the ear. In the organ of Corti, reduction and delayed deletion of *Atoh1* result in progressive loss of almost all the inner hair cells and the majority of the outer hair cells within three weeks after birth. The remaining cells express hair cell marker Myo7a and attract nerve fibers, but do not differentiate normal stereocilia bundles. Some Myo7a-positive cells persist in the cochlea into adult stages in the position of outer hair cells, flanked by a single row of pillar cells and two to three rows of disorganized Deiters cells. Gene expression analyses of *Atoh1, Barhl1* and *Pou4f3*, genes required for survival and maturation of hair cells, reveal earlier and higher expression levels in the inner compared to the outer hair cells. Our data show that *Atoh1* is crucial for hair cell mechanotransduction development, viability, and maintenance and also suggest that *Atoh1* expression level and duration may play a role in inner vs. outer hair cell development. These genetically engineered *Atoh1* CKO mice provide a novel model for establishing critical conditions needed to regenerate viable and functional hair cells with *Atoh1* therapy.

## Introduction

Sensorineural hearing loss is one of the most common sensory disorders and mostly results from loss of cochlea hair cells in the inner ear. To understand the pathology and develop therapeutic strategies to restore hearing, numerous attempts have therefore been focused on using proper animal models to test various approaches to regenerate hair cells, including transdifferentiating supporting cells into hair cells [1], [2], [3] or transforming multipotent stem cells into hair cells [4], [5], [6], [7].

The most frequently manipulated gene in these studies is *Atoh1*, a proneural basic helix-loop-helix (bHLH) transcription factor that is essential for inner ear hair cell differentiation [8]. Knockout of *Atoh1* in mice results in complete absence of differentiated hair cells [8], [9]. The majority of the hair cell precursors undergo cell death and the organ of Corti is transformed into a flat epithelium in postnatal stage [9], [10]. Previous work has also shown that misexpression of *Atoh1* in tissue culture or *in vivo* at various stages can induce extra hair cell formation [11], [12]. However, the level and duration of *Atoh1* expression required for normal hair cell development and successful regeneration of viable hair cells has not been determined. It remains unclear if some differentiation might be possible with limited or transient expression of *Atoh1*.

To directly test the degree of hair cell differentiation with limited and transient *Atoh1* expression as typically achieved with *Atoh1* therapy [11], [12], we developed a novel strategy to delete *Atoh1* with a delay. It has been shown that *Atoh1* autoregulates its expression by binding to an E-box consensus site in a highly conserved enhancer that has been shown to be sufficient to drive *Atoh1* specific gene expression in transgenic mice [13]. Previously we have generated a *Tg(Atoh1-cre)* line using the *Atoh1* enhancer region and showed the expression of *cre* in *Atoh1* expression domains, including the inner ear hair cells [14]. By crossing *Tg(Atoh1-cre)* with a mouse line carrying the floxed *Atoh1* gene [15], we generated a unique *Atoh1* conditional knockout (CKO) mouse line, in which Atoh1 protein ‘self-terminates’ its expression by upregulating the expression of *cre*, resulting in transient, limited expression of *Atoh1* in all hair cells. Characterization of this CKO mouse line indicates that almost all inner hair cells are lost quickly through cell death, while some outer hair cells can continue to express hair cell markers such as Myo7a and survive into adulthood. Our data suggest that proper *Atoh1* expression level and duration is required for the viability and differentiation of hair cells.

## Materials and Methods

### Breeding, genotyping and collection of mice

All animal work was conducted according to the Guide to the Care and Use of Laboratory Animals and all procedures were approved by the University of Iowa Institutional Animal Care and Use Committee (IACUC) (ACURF #1103057).

To generate the *Atoh1-cre;Atoh1^f/f^* conditional knockout (CKO) mice, we bred the mice carrying the *Atoh1-cre* transgene [14] with mice carrying the floxed *Atoh1* [15], [16]. The CKO mutants are viable and were obtained at the expected Mendelian ratio at all stages. Littermates with the genotypes of *Atoh1-cre;Atoh1^f/+^* or *Atoh1^f/f^* were used as controls.

The mice were genotyped using PCR analysis of the tail DNA. The *Atoh1-cre* transgene was detected by *cre*-specific primers (forward: 5′-CCT GTT TTG CAC GTT CAC CG-3′ and reverse: 5′-ATG CTT CTG TCC GTT TGC CG-3′), which produced a 280 bp product. Two internal control primers (forward: 5′-CTA GGC CAC AGA ATT GAA AGA TCT-3′ and reverse: 5′- GTA GGT GGA AAT TCT AGC ATC ATC C-3′) were included in the PCR reaction, which produced a 330 bp product. The *Atoh1* alleles were distinguished using two primers (forward: 5′-AGC GAT GAT GGC ACA GAA G-3′ and reverse: 5′-GAA GTC AAG TCG TTG CTA AC-3′). The PCR product sizes are 300 bp for the wild-type *Atoh1* allele and 500 bp for the floxed allele.

All postnatal mutant and control mice or pregnant females for collecting embryos were anesthetized by injection of 0.025 ml/g of body weight of Avertin (1.25% of 2.2.2-tribromoethanol) and then were perfused with 4% paraformaldehyde (PFA) in 0.1 M phosphate buffer (pH 7.4) using a peristaltic pump. Heads were isolated and fixed in 4% PFA for at least 24 hours. The ears from animals older than P7 were decalcified in saturated EDTA in 0.4% PFA before being dissected for further processing.

### *In situ* hybridization

*In situ* hybridization was carried out according to Duncan et al. [17]. The antisense RNA probes were generated from cDNA-containing plasmids and labeled with digoxigenin by *in vitro* transcription using DIG RNA labeling kit (Roche Applied Science, Cat. 11175025910). Whole mount *in situ* hybridization was performed on mutant and corresponding control ears from opposite sides simultaneously. The PFA fixed and dissected ears were digested briefly with 20 µg/ml of Proteinase K (Ambion, Cat. AM2546) for 15–20 minutes and then hybridized overnight at 60°C to the probe in hybridization solution containing 50% (v/v) formamide, 50% (v/v) 2X saline sodium citrate and 6% (w/v) dextran sulphate. After washing off the unbound probe, the ears were incubated overnight with an anti-digoxigenin antibody conjugated with alkaline phosphatase (Roche Applied Science, Cat. 11093274910). After a series of washes, the samples were reacted with 4-nitro blue tetrazolium chloride (NBT)/5-bromo-4-chloro-3-indolyl-phosphate (BCIP) (BM purple substrate, Roche Applied Science, Cat. 11442074001), which is enzymatically converted to a purple colored product. The ears were then flat mounted in glycerol and viewed in a Nikon Eclipse 800 compound microscope using differential interference contrast microscopy and images were captured with Metamorph software.

For sections of the embryonic day 14.5 (E14.5) ears, the reacted ears were dehydrated in graded ethanol, immersed in propylene oxide, embedded with Epon 812 in beam capsules and polymerized at 60°C for at least 24 hours. Three µm thick sections were cut using a Leica RM2265 Microtome with glass knives, mounted on slides, lightly stained with Stevenel's blue and viewed using a Nikon Eclipse 800 microscope.

### Immunohistochemistry and apoptotic cell labeling

For immunohistochemical staining, the ears were first dehydrated in 70% ethanol and after rehydration with graded ethanol and PBS were blocked with 2.5% normal goat serum in PBS containing 0.25% Triton X-100 for 1 hour. One or a combination of the primary antibodies for activated Caspase 3 (1∶100; Cell Signaling, 9661), Cre (1∶1000, Covance PRB-106C), Espin (1∶5, a gift from Dr. James Bartles), Myo7a (1∶200; Proteus Biosciences, 25-6790), and Tubulin (1∶800; Sigma, T7451) were added and incubated for 48 hours at 4°C. After several washes with PBS, corresponding secondary antibodies (1∶500; Molecular Probes Alexa fluor 647, 532, or 488; Invitrogen) were added and incubated overnight at 4°C. The PSVue480 (10 µM; Molecular Targeting Technologies, Inc., P-1003) and Hoechst (5 µg/mL; Polysciences, Inc., 09460) dyes were then added and incubated for 15 minutes at room temperature. The ears were washed with PBS and mounted in glycerol and images were taken with a Leica TCS SP5 confocal microscope using appropriate excitation/emission settings.

For sections of the P38 ears, the reacted ears were embedded and sectioned at five µm thickness and images were taken with a Leica TCS SP5 confocal microscope. The control ears were sectioned at three µm thickness, stained with Stevenel's blue, and viewed using a Nikon Eclipse 800 microscope.

To combine immunohistochemistry and *in situ* hybridization, we pretested selective antibodies to verify that they still recognize the epitopes after proteinase K digestion and performed immunohistochemistry after the *in situ* hybridization. After the fluorescence and bright field images were taken from the same field, we false-colored the *in situ* signal and combined with the immunofluorescence images using ImagePro software.

### Scanning electron microscopy (SEM)

Dissected (and decalcified for P21) ears were post-fixed in 2.5% glutaraldehyde overnight, followed by rinsing in 0.1 M phosphate buffer (pH 7.4) and secondary fixation with 1% osmium tetroxide in 0.1 M phosphate buffer. Osmicated ears were washed several times in distilled water to remove all ions, dehydrated in a graded ethanol series and further dehydrated using a critical point dryer. The ears were then mounted on stubs with carbon tapes and coated with gold/palladium using a K550 Emitech sputter coater at 10 mA for three minutes. Samples were viewed with a Hitachi S-4800 SEM using a 10 µA emission current.

### Auditory brainstem response (ABR) recording

Three month old control and mutant mice were first injected with Avertin (0.025 ml/g of body weight) to induce a surgical level of anesthesia, as assessed by absence of ocular and pedal reflexes. Then needle electrodes were inserted in the vertex, slightly posterior to the pinna and in the contralateral hind limb subcutaneously. A loud speaker was placed 10 cm from the pinna of the test ear and computer-generated clicks were given in an open field environment in a soundproofed chamber. Clicks were presented and responses were averaged across 512 presentations using Tucker-Davis Technologies System hardware running BioSig® Software. Recorded signals were bandpass filtered (300 Hz–5 kHz) and 60Hz notch filter. The sound level was decreased in 10-dB steps from a 96-dB sound pressure level until there was no noticeable response.

## Results

### *Atoh1-cre* limits the initial *Atoh*1 expression and eliminates it after a few days in all hair cells

**Figure 1:**
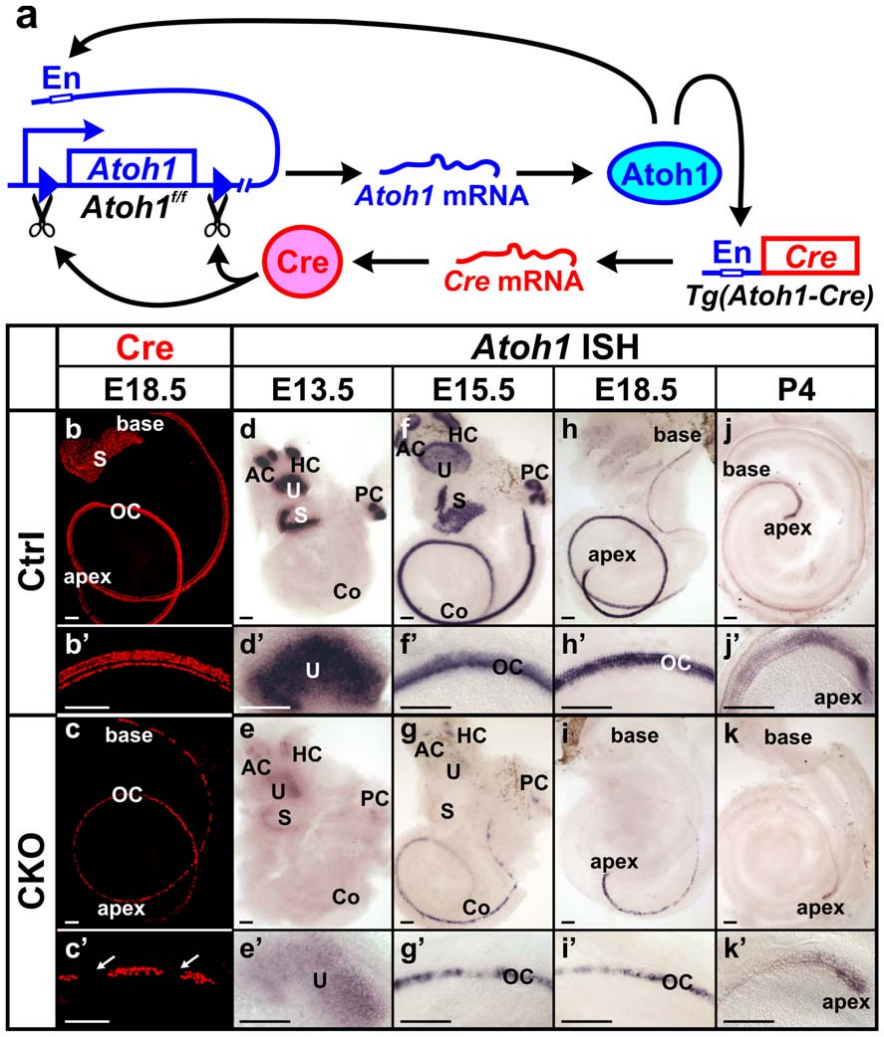
*Atoh1-cre* is expressed in hair cells and causes transient limited expression of *Atoh1* in CKO ears. (**a**) This diagram shows that *Atoh1-cre* is driven by an *Atoh1* enhancer element (En) which is activated when endogenous Atoh1 protein binds. After the Cre protein is expressed, it excises the genomic *Atoh1* region flanked by loxP sites, resulting in conditional deletion of *Atoh1* after a transient expression of mRNA and protein. (**b–c’**) Immunohistochemistry of Cre protein (red) at E18.5 confirms that Cre is expressed in all hair cells in the control ear. The expression in the CKO mutant cochlea is limited to one to two rows of cells in the organ of Corti and becomes discontinuous with gaps (arrows in c’) in the base. (**d–k’**) *In situ* hybridization of *Atoh1* shows its expression in the control and CKO mutant ears. The expression initiates in vestibular sensory epithelia (d) followed by progressive expression from the base to the apex in the cochlea (f, h, j). In the CKO ears *Atoh1* expression is less profound and later is eliminated in all sensory epithelia (e, g, i, k). The loss of *Atoh1* in the CKO cochlea also progresses from base to apex. At P4, only a small number of cells at the apical tip show *Atoh1* expression (k, k’). All control and mutant ears of the same stages are from littermates processed simultaneously as left and right ears for identification. Images were electronically adjusted to the same orientation. AC, anterior crista; Co, cochlea; HC, horizontal crista; OC, organ of Corti; PC, posterior crista; S, saccule; U, utricle. Bar indicates 100 µm.

Previously we have reported the generation of an *Atoh1-cre* transgenic mouse line, in which an *Atoh1* enhancer fragment [10], [13] was used to drive *cre* expression [14]. The activation of this enhancer is dependent on the Atoh1 protein, which binds to the enhancer and upregulates both endogenous *Atoh1* expression and *cre* expression from the transgene and can thus ‘self-terminate’ its expression through Cre mediated recombination (Fig. 1a). Previous studies using the R26R reporter line has shown that *Atoh1-cre* is activated in all inner ear hair cells [14]. In E18.5 *Atoh1-cre;Atoh1^f/+^* animals, which contain the *cre* transgene and only one allele of floxed *Atoh1*, we carried out immunohistochemistry of Cre and found Cre protein in the hair cells of all vestibular endorgans and the cochlea (Fig. 1b, b’). This confirmed that the *Tg(Atoh1-cre*) expression pattern follows endogenous *Atoh1* expression. In E18.5 *Atoh1-cre;Atoh1^f/f^* CKO mutants, the Cre-expressing cells were reduced in all sensory epithelia. In contrast to one row of inner and three rows of outer hair cells in the control cochlea, only one to two rows of cells were positive for Cre in the mutant cochlea and there were variable-sized gaps between Cre-positive cells predominantly in the base of the cochlea (Fig. 1c, c’).

To assess the recombination of floxed *Atoh1* by Cre, we performed *in situ* hybridization of *Atoh1* at different developmental stages. In E13.5 control ears, *Atoh1* mRNA was detected in all five vestibular endorgans (Fig. 1d, d’). The mutant littermates also displayed *Atoh1* expression in all vestibular epithelia, but the level was greatly reduced (Fig. 1e, e’), suggesting that the process from Atoh1 protein activating *cre* expression to Cre recombining floxed *Atoh1* is rather quick and limits generating the full expression level of *Atoh1* mRNA. This almost immediate decrease of *Atoh1* expression was also obvious in the mutant cochlea, where normal expression starts at the base progressing towards the apex. At E15.5, in contrast to the strong expression of *Atoh1* in the control cochlea (Fig. 1f, f’), *Atoh1* signal in the mutant was narrower, much weaker, and became discontinuous at the base (Fig. 1g, g’). At E18.5, the discontinuity of *Atoh1* expression progressed to the upper middle turn of the mutant cochlea and the base showed no detectable *Atoh1* expression (Fig. 1i, i’). Only at the region close to the apical tip of the cochlea, where the normal *Atoh1* expression reaches at this stage (Fig. 1h, h’), the expression in the mutant was still continuous but with reduced intensity. *Atoh1* expression remained in the apex of the mutant cochlea at least until P4 (Fig. 1k, k’), consistent with the normal expression in control cochlea that diminished in a base to apical gradient (Fig. 1j, j’). Our data showed that the *Atoh1-cre* transgene could effectively delete the floxed *Atoh1* gene with little delay following its expression, resulting in limited *Atoh1* expression followed by loss of *Atoh1* mRNA within a few days after the initial expression in all inner ear hair cells.

### Progressive loss of *Atoh1* leads to patchy distribution of hair cells in the *Atoh1* CKO neonate ear

**Figure 2:**
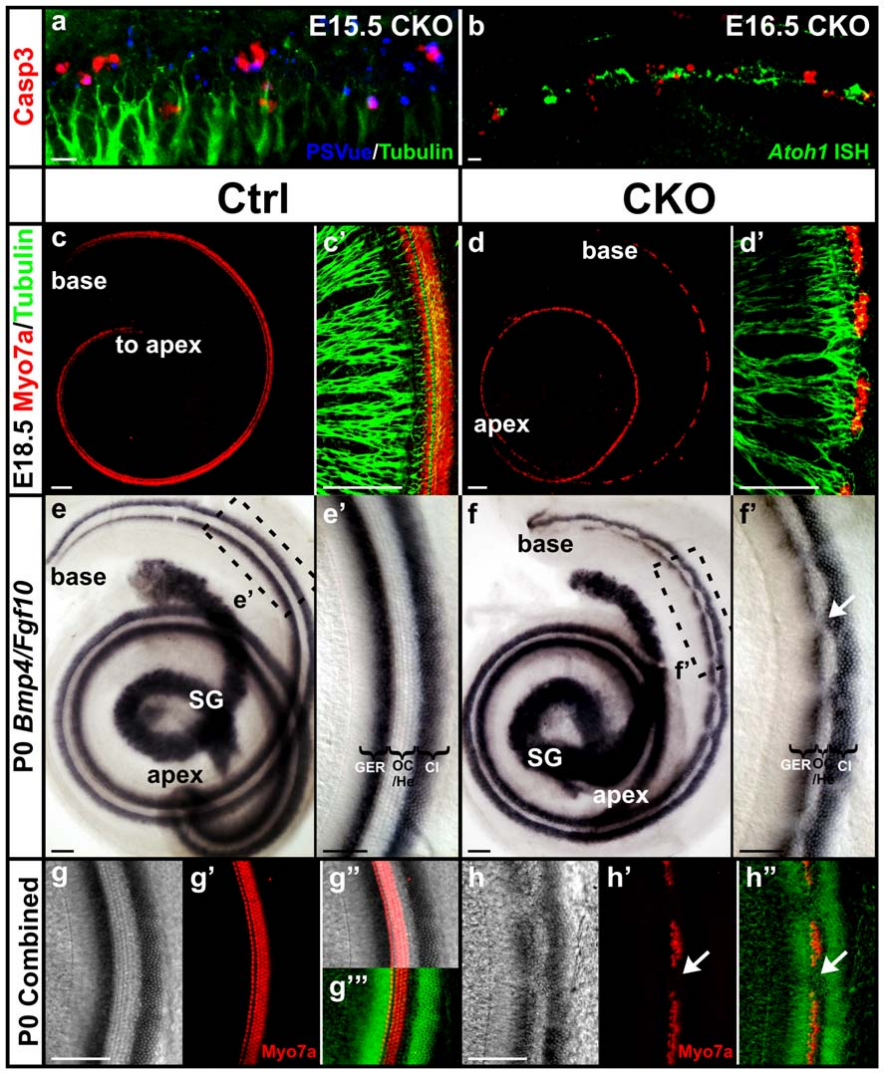
Conditional deletion of *Atoh1* results in death of organ of Corti cells and patchy Myo7a-positive presumptive hair cells which are innervated by many nerve fibers. (**a–b**) Cell death assay using immunohistochemistry of activated Caspase 3 (red) and PSVue staining (blue) shows that many cells undergo cell death in E15.5 and E16.5 CKO ears. Whole mount cochlea is shown with the lateral wall to the top and the modiolus to the bottom. Dying cells are seen in the region equivalent to the organ of Corti based on their location in close proximity to the nerve fibers shown by Tubulin staining (green in a). When combined with *Atoh1 in situ* hybridization (false-colored in green in b), many Caspase 3-positive dying cells are found in between the *Atoh1*-expressing cells (b). (**c–d’**) Myo7a and Tubulin immunostaining shows presumptive hair cells and their innervation in E18.5 cochlea. In contrast to the stereotyped one row of inner and three rows of outer hair cells seen in control cochlea (c’), the Myo7a-positive hair cells are reduced to only one to two rows in the CKO cochlea (d’). They are continuous in the apex but become patchy in the base with gaps in between (d, d’). The remaining hair cells apparently attract most of the nerve fibers that innervate the mutant ear. (**e–f’**) Double *in situ* hybridization for *Bmp4* and *Fgf10* shows the boundaries of the organ of Corti. *Bmp4* is expressed in Claudius cells lateral to the organ of Corti. *Fgf10* is expressed in the greater epithelial ridge and defines the medial boundary. In the CKO cochlea, the two expression regions are in parallel in the apex (f) like the control (e). However, the *Fgf10* expression is discontinuous at the base and the *Bmp4* expression is expanded towards the gaps in the *Fgf10* expression (f, f’). (**g–h”**) Immunostaining of Myo7a is combined with the *Bmp4*/*Fgf10* double *in situ* hybridization (false-colored in green in g’” and h”) and shows that the Myo7a-positive hair cells are located between *Bmp4* and *Fgf10* expression regions. In the base of the CKO cochlea, the gaps between Myo7a-positive cells correlate with the area where *Bmp4* expression is expanded towards GER (arrows in h’ and h”). Cl, Claudius cells; GER, greater epithelial ridge; He, Hensen’s cells; OC, organ of Corti; SG, spiral ganglion. Bar indicates 10 µm in a, b and 100 µm in c–h”.

Our previous study using a *Pax2-cre* transgene to conditionally delete *Atoh1* showed that in the absence of *Atoh1*, most of the organ of Corti cells were lost through cell death [9], confirming earlier suggestions in *Atoh1* null mice [10], [18]. We analyzed the cell death in *Atoh1-cre;Atoh1^f/f^* CKO mutants using an antibody against activated Caspase 3 and the PSVue dye, which specifically binds to phosphatidylserine (PS) exposed on the membranes of dying cells [19]. At E15.5, both control and mutant ears displayed a few dying cells in the developing spiral ganglia (data not shown). However, we found many apoptotic cells in the region topographically equivalent to the organ of Corti only in the mutant ear (Fig. 2a). To further determine how the degeneration of the organ of Corti cells is related to the loss of *Atoh1* expression in hair cells, we investigated the distribution of dying cells after *Atoh1 in situ* hybridization in E16.5 ears. At this stage, the *Atoh1* expression was already discontinuous at the base of the mutant cochlea. Interestingly, most Caspase 3-positive cells were not coinciding with the *Atoh1*-expressing cells (Fig. 2b), suggesting that the loss of *Atoh1* may trigger cell death of these hair cells and, possibly with some delay, of surrounding supporting cells.

To determine the effect of the *Atoh1* loss on hair cells, we next examined the hair cell marker Myo7a in mutant ears. At E14.5, Myo7a-positive cells were present in the vestibular sensory epithelium in both control and mutant ears (Fig. S1a–d’). However, the distribution of Myo7a-positive cells became sporadic at E18.5, in particular in the saccule (Fig. S1f, f’). In the mutant cochlea the Myo7a-positive cells were also reduced from four rows to only one to two rows. These cells were continuous at the apical half but became discontinuous towards the base (Fig. 2d, d’), which was consistent with the pattern we found with Cre immunostaining (Fig. 1c, c’). This indicated that the low levels of *Atoh1* mRNA shown by *in situ* hybridization generated enough Atoh1 protein to activate the *Atoh1-cre* transgene in viable hair cell precursors. These cells also attracted the majority of the nerve fibers from the spiral ganglion neurons (Fig. 2d’), although the total innervation seemed to be decreased compared with the control ear (Fig. 2c’ d’, S1e’, f’). Thus, in *Atoh1-cre;Atoh1^f/f^* CKO mice, some hair cells were lost via cell death and the remaining hair cells differentiated to the extent that they expressed Myo7a and Cre protein following a limited transient *Atoh1* expression.

To further evaluate the development of the organ of Corti, we investigated the expression of two genes, *Fgf10* and *Bmp4*, which define the boundaries of the organ of Corti even in the complete absence of hair cells in a *Pax2-cre Atoh1* CKO mutant [9], [20], [21]. *Bmp4* is expressed in the Claudius cells lateral to the organ of Corti and *Fgf10* is expressed in the greater epithelial ridge (GER) medial to the organ of Corti and in spiral ganglion cells. In P0 control cochlea, double *in situ* hybridization of these two genes showed two parallel expression regions flanking the organ of Corti (Fig. 2e, e’). However in the mutant littermate, the expression of *Fgf10* was patchy in the basal half of the cochlea and *Bmp4* expression expanded towards the GER in between the gaps of *Fgf10* expression (Fig. 2f, f’). In the apex, the two expression regions were in parallel (Fig. 2f).

To directly correlate the expression of these two flanking markers with the remaining hair cells, we performed Myo7a immunohistochemistry on the *in situ* hybridized ears and combined the images by false-coloring the *in situ* hybridization signal (Fig. 2g”, g’”, h”). We found that the area where *Fgf10* expression was absent and *Bmp4* expanded towards GER correlated with the gaps between Myo7a-positive cells (Fig. 2h”). It was unclear if these epithelial Claudius cell-like cells were dedifferentiated hair cells that adopt the Claudius cell fate or simply reflected the expansion of Claudius cells.

### *Atoh1* affects hair cell stereocilia development and maintenance

**Figure 3:**
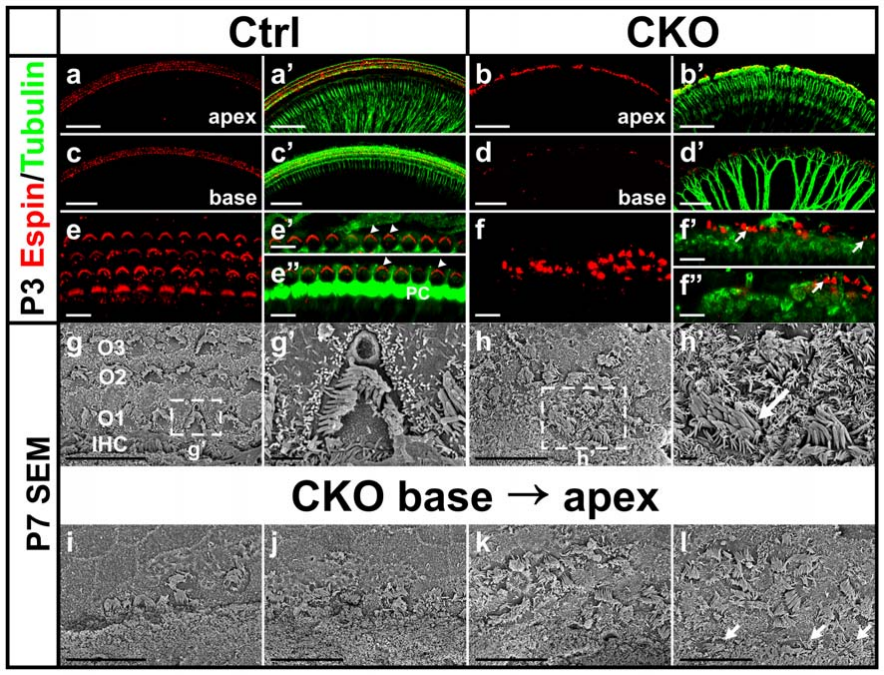
The remaining hair cells in the *Atoh1* CKO ears develop abnormal stereocilia. (**a–f”**) Immunohistochemistry of Espin (red) shows the stereocilia bundles in the hair cells in P3 ears. The control hair cells have well-ordered stereocilia in both inner and outer hair cells (a, c, e). In green, the Tubulin antibodies label nerve fibers, supporting cells and the kinocilia located off-center of the stereocilia bundle (arrowheads in e’, e”). In the CKO cochlea, the Espin-positive cells are reduced to only one to two rows and become discontinuous towards the base (b, d). The stereocilia bundles are misshaped with kinocilia at abnormal positions (arrows in f’, f”). e and f show all the Espin-positive cells by collapsing z-stack confocal images while e’, e”, f’ and f” are single optical slices. (**g–h’**) SEM images of P7 control and CKO cochlea hair cells. High magnification images of several CKO hair cells (h’) shows aberrant stereocilia compared to the control (g’). Note that one inner hair cell-like cell has unusually thick stereocilia (arrow in h’). (**i–l**) A series of images taken from base to the apex of the CKO organ of Corti show that hair cells are progressively lost along the length of the cochlea. There are only small patches of hair cells in the base, while two to three rows of outer hair cells and one row of inner hair cells (arrows in l) remain in the apical tip. However, the stereocilia of all these hair cells are abnormal compared to littermate control (g). IHC, inner hair cells; O1, O2, O3, three rows of outer hair cells; PC, pillar cells. Bar indicates 100 µm in a-d’, 10 µm in e-g, h, i-l, and 1 µm in g’, h’.

Mechanotransduction is the fundamental characteristic of differentiated hair cells. We therefore investigated if the Myo7a-positive cells in the *Atoh1-cre;Atoh1^f/f^* CKO mice developed normal stereocilia needed for mechanotransduction using an antibody against Espin, an actin-bundling protein that is expressed in the hair cell stereocilia [22], [23]. In P3 control cochlea, Espin immunolabeling showed C-shaped stereocilia bundles in all the inner hair cells and W-shaped stereocilia bundles in outer hair cells (Fig. 3a, c, e). The Tubulin antibody labeled the supporting cells and the kinocilia positioned off-center of the stereocilia bundle (Fig. 3e’, e”). In the cochlea of the mutant littermate, we found Espin-positive cells in one to two rows in the apex but even fewer cells in the base (Fig. 3b, d, f). The hair bundles were irregularly shaped with kinocilia at odd positions (Fig. 3f’, f”). In addition, the mutant also displayed abnormal hair bundles and polarity in the vestibular hair cells (Fig. S2b’, e, f), occasionally showing no stereocilia associated with kinocilia (arrows in Fig. S2f). These data suggest that a normal level of *Atoh1* is important for the proper development of the hair bundles and their polarity.

Tubulin immunolabeling also showed densely spaced radial fiber bundles toward hair cells in the control (Fig. 3a’, c’). The radial bundles of nerve fibers were more widely spaced in the mutant, concentrating near the remaining Espin-positive cells (Fig. 3d’). The reduced density of nerve fibers near gaps was consistent with data on diminished innervation in *Atoh1* null mice [9], [18] and suggested that neurotrophic support [24] was diminished in the gaps between the remaining hair cells.

We also used scanning electron microscopy (SEM) to directly examine the stereocilia development of the hair cells. In P7 mutant ears, we found that some cells in the position of the organ of Corti developed stereocilia-like structures but they were disorganized and misshapen (Fig. 3h, h’). The number of hair cell-like cells progressively increased from base (Fig. 3i) to apex (Fig. 3j, k). At the apical tip up to four rows of hair cells were seen, with the most medial row of cells exhibiting inner hair cell-like thicker stereocilia (Fig. 3l). However, very few cells displayed normal stereocilia organization and orderly polarity as in the control littermates (Fig. 3g, g’). In the base, the gaps between the small patches of hair cells were occupied by epithelial cell-like cells, consistent with the expansion of Claudius cell-like cells positive for *Bmp4 in situ* hybridization (Fig. 2f, h, h”).

SEM of a P7 mutant saccule also showed decreased number of hair cells and abnormal stereocilia development (Fig. S2h-h”). Different from the systemic *Atoh1* null [25] and the *Pax2-cre Atoh1* CKO [9], the *Atoh1-cre;Atoh1^f/f^* CKO animals had no severe breathing or general growth defects and can live well into adulthood, allowing us to analyze long-term effects of delayed conditional *Atoh1* loss. In an 11-month old mutant mouse, we found very long, irregular spaced stereocilia in the remaining posterior crista hair cells (Fig. S2i–i’”). Consistent with the abnormalities in vestibular hair cells, we noted abnormal motor behavior including head shaking and irregular walking patterns, suggesting some vestibular and also possibly cerebellar dysfunctions in these mutant animals.

**Figure 4:**
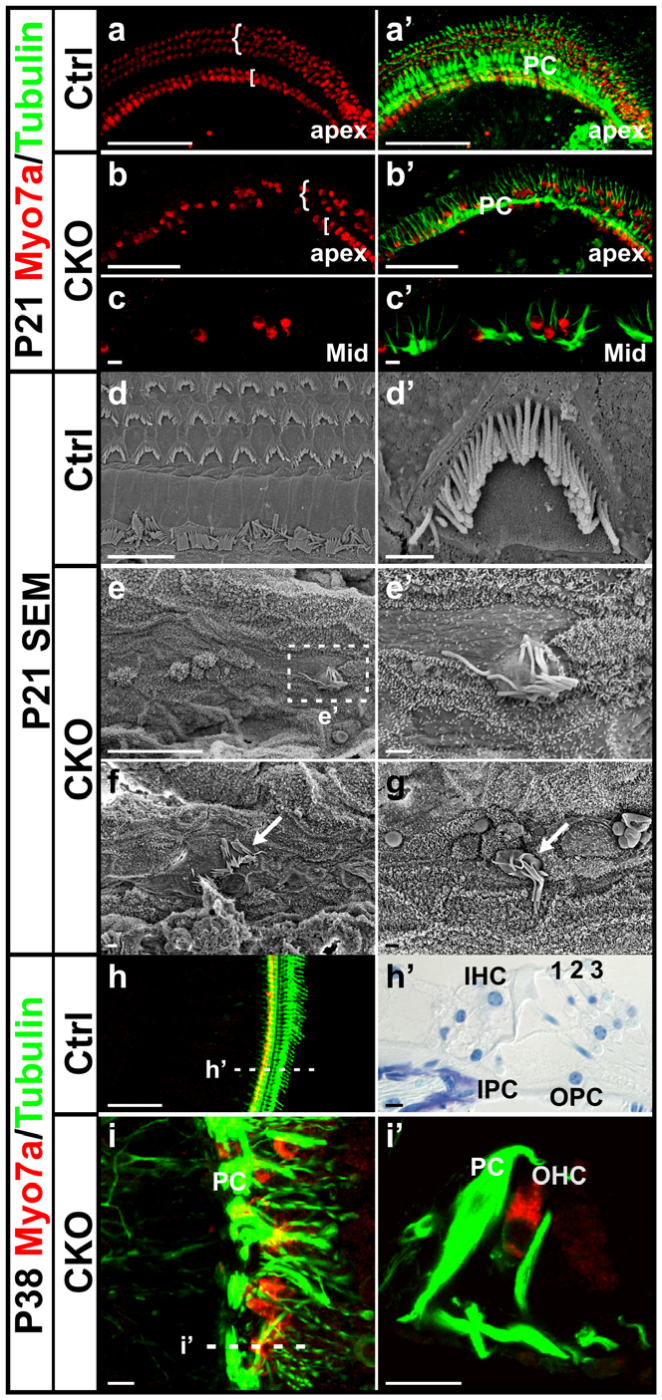
Some Myo7a-positive cells persist in adult *Atoh1* CKO ears and display abnormal stereocilia. (**a–c’**) In P21 control cochlea, one row of inner hair cells and three rows of outer hair cells are labeled by Myo7a (red) and the supporting cells are labeled by Tubulin (green). The pillar cells between the inner and outer hair cells are most strongly labeled. In the CKO cochlea, a few inner hair cells, which are medial to the strongly Tubulin-labeled pillar cells, can only be found at the apical tip (b, b’). The outer hair cells are reduced to one to two rows in the apex but become scattered in the middle turn and are surrounded or next to the remaining Tubulin-positive supporting cells. (**d–g’**) In contrast to the highly organized stereocilia found in all hair cells throughout the control cochlea, only few cells in the CKO cochlea are found to be hair cell-like, with stereocilia-like structures (e’, arrows in f, g). They are surrounded by, next to, or completely isolated from supporting cell-like cells. (**h–i’**) Immunohistochemistry of Myo7a and Tubulin shows hair cells and supporting cells in P38 ears. The control ear shows characteristic cellular distribution throughout the cochlea (h, h’). However, only a few Myo7a-positive cells remain in the CKO cochlea apex (i). The section shows that this hair cell is lateral to a Tubulin-labeled pillar cell-like cell and thus in the position of the first row of outer hair cells (i’). ‘[‘ and ‘{‘ in a and b indicate the location of inner and outer hair cells respectively. The dotted lines in h and i indicate the approximate position of the sections shown in h’ and i’. 1, 2, 3, three rows of outer hair cells; IHC, inner hair cells; IPC, inner pillar cell, OPC; outer pillar cell; PC, pillar cells. Bar indicates 100 µm in a–b’, h, 10 µm in c, c’, d, e, h’, i, i’, and 1 µm in d’ e’, f, g.

To determine the long-term viability of the remaining hair cells and surrounding supporting cells in the mutant cochlea, we examined P21 ears using Myo7a and Tubulin immunohistochemistry. All the hair cells and supporting cells in the control ear continued expressing Myo7a and Tubulin, respectively (Fig. 4a, a’). In contrast, we found a cluster of Myo7a-positive cells only in the apex of the mutant cochlea, the majority of which were in the position of outer hair cells, lateral to a single row of strongly Tubulin-labeled pillar cells (Fig. 4b, b’). Only a few Myo7a-positive cells at the apical tip appeared to be in the position of inner hair cells, consistent with our early stage SEM data. In the middle turn and base of the mutant cochlea, only individual or small groups of Myo7a-positive cells were found (Fig. 4c). They were usually surrounded by cells showing Tubulin immunolabeling, but occasionally were also seen at a distance from Tubulin-positive cells (Fig. 4c’). When we examined the P21 ears using SEM, we only found a few individual cells with stereocilia-like structures (Fig. 4e–g). They were in the topographical position of the organ of Corti and a few cells either surrounding or close by these cells showed dense tall microvilli on the surface (Fig. 4e-g).

To determine the fate of these cells, we analyzed P38 ears by whole mount immunohistochemistry followed by sectioning. Histologic staining of the control cochlea sections showed the stereotyped one row of inner and three rows of outer hair cells and also the well-organized supporting cells (Fig. 4h’). In the mutant cochlea, only a few Myo7a-positive cells were found in the apex and they were located lateral to the single row of Tubulin-labeled pillar cells (Fig. 4i, i’). We also examined P88 mutant ears using SEM but did not find any hair cell-like cells. The organ of Corti appeared as a flat epithelium (data not shown). Consistent with these data, the mutant animals were deaf shown by the complete lack of ABR (auditory brainstem response) when tested at three months of age (Fig. S3). The further reduction of hair cells in older mutant animals suggested that continued expression of *Atoh1,* previously demonstrated in adult hair cells using the *Atoh1-LacZ* reporter [14], may play a role in long-term maintenance of hair cells.

### *Atoh1*, *Barhl1* and *Pou4f3* are differentially regulated in the inner and outer hair cells

**Figure 5:**
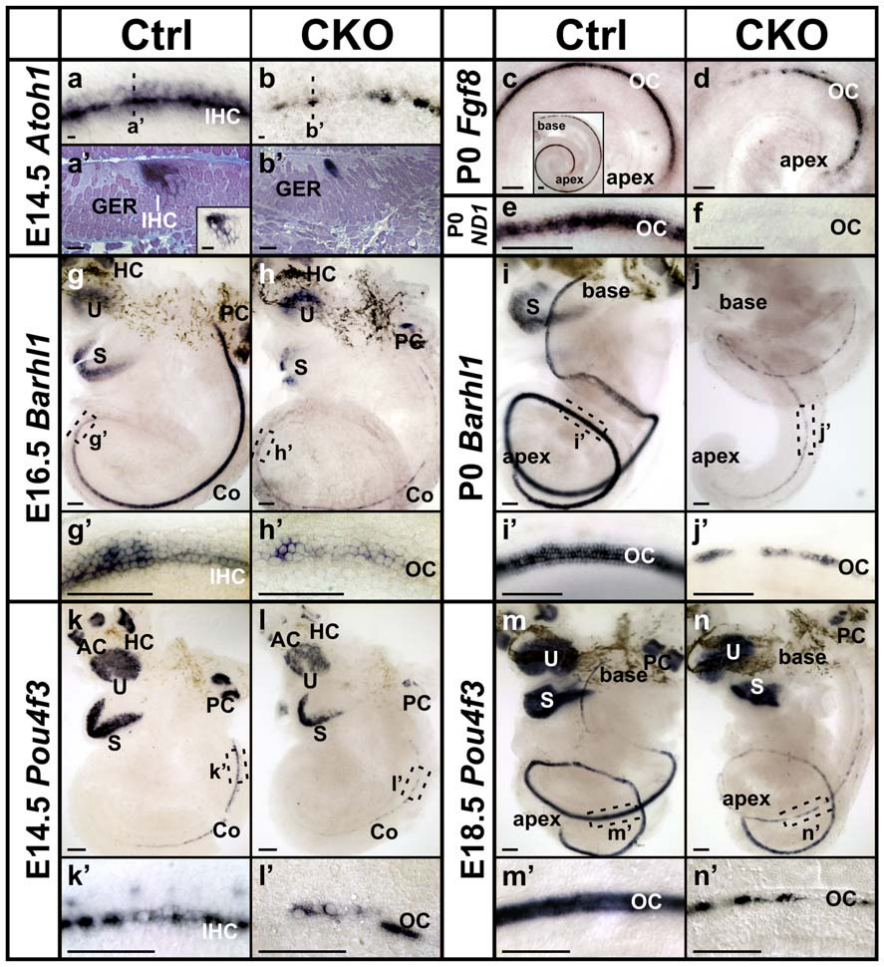
*Atoh1, Fgf8* and *Pou4f3,* and to some extent *Barhl1,* are differentially expressed in inner and outer hair cells. (**a–b’**) *Atoh1 in situ* hybridization in E14.5 ears shows its early expression in the cochlea. Both whole mount (a) and sections (a’) of control cochlea show a gradient of *Atoh1* expression, with stronger signal in the inner hair cells and lower level in the outer hair cells. The tissue in the sections is stained (purple) and the *Atoh1 in situ* signal is dark blue. Note in unstained sections, low levels of *Atoh1* signal can also be seen in several cells around the hair cells, possibly the supporting cells (insert in a’). In CKO cochlea, the *Atoh1* signal appears sporadic in single or two rows of hair cells (b, b’). (**c–d**) *Fgf8* is specifically expressed in the inner hair cells in the entire P0 control cochlea but only in the apex of the CKO cochlea. (**e–f**) *Neurod1* is expressed and acts downstream of *Atoh1* in the hair cells. *In situ* hybridization shows there is a complete loss of *Neurod1* in P0 CKO cochlea. (**g–j’**) *Barhl1*, an *Atoh1* downstream gene, follows its base to apex expression in the hair cells in the ear. At the leading end in E16.5 control cochlea, *Barhl1* is first upregulated in the inner hair cells (g’). The expression is greatly reduced and becomes intermittent in the mutant ear (h, h’). At P0, all hair cells express *Barhl1* in the control ear, but the expression is further reduced in the CKO cochlea. (**k–n’**) *In situ* hybridization of *Pou4f3* shows its expression in hair cells. In E14.5 control cochlea, the expression starts from the base and the inner hair cells. Only a few outer hair cells express *Pou4f3* at this stage (k’). At E18.5, the expression levels in all hair cells are similar. In CKO ears at both stages, the expression of *Pou4f3* is greatly reduced, except for the apex. AC, anterior crista; Co, cochlea; GER, greater epithelia ridge; HC, horizontal crista; IHC, inner hair cells; OC, organ of Corti; PC, posterior crista; S, saccule; U, utricle. Bar indicates 10 µm in a-b’ and 100 µm in c–n’.

To elucidate the difference of viability between inner and outer hair cells, we further investigated the early expression of *Atoh1* at E14.5 and found a gradient with the highest expression level in the inner hair cells and lower level in the outer hair cells in both whole mount and sections of control cochlea (Fig. 5a, a’). The similar medial to lateral gradient was also seen in the apex of E15.5 control cochlea (Fig. 1f, f’), where the *Atoh1* expression is initiated at this stage. In E14.5 CKO mutant cochlea, the *Atoh1* expression area was already narrowed and became intermittent. The residual *Atoh1* signal appeared only sporadic in inner hair cells (Fig. 5b, b’), suggesting early loss of inner hair cells in the mutant.

*Fgf8* has been shown to be specifically expressed in the inner hair cells starting from E16 at the base [26], [27]. At P0, *in situ* hybridization showed *Fgf8* expression in all the inner hair cells in control cochlea (Fig. 5c insert). In *Atoh1-cre;Atoh1^f/f^* CKO ears, we found *Fgf8* expression was restricted to the apex of the cochlea (Fig. 5d) as previously reported in *Atoh1* null mice [28]. Compared with the continuous apical and patchy basal Myo7a immunolabeling at P0 (Fig. 2h’, data not shown), this further suggested the early loss of almost all the inner hair cells and longer retention of some outer hair cells in the mutant.

We next examined the expression of *Neurod1*, which is essential for sensory neuron survival [29] but has also been shown to be expressed and act downstream of *Atoh1* in the hair cells [14], [30]. At P0, *in situ* hybridization of *Neurod1* shows its expression in the control cochlea but a complete loss in the mutant (Fig. 5e, f), suggesting that a sustained and high level of *Atoh1* expression is required for *Neurod1* expression.

*Barhl1* is a homeobox gene essential for the long-term maintenance of cochlea hair cells [31] and has been shown to be regulated by *Atoh1* [32]. Its expression also followed the base to apex progression. Shown by *in situ* hybridization, *Barhl1* expression reached about three quarters of the E16.5 control cochlea (Fig. 5g). At the apical leading edge, we detected *Barhl1* expression only in the inner hair cells (Fig. 5g’), which is consistent with a previous study at E18.5 apex [32]. In littermate CKO cochlea, the *Barhl1* expression appeared delayed and greatly reduced to patches (Fig. 5h, h’). At P0, in contrast to the strong expression in the control cochlea (Fig. 5i, i’), only residual patchy expression of *Barhl1* were detected in the mutant cochlea (Fig. 5j, j’).

We next investigated the expression of *Pou4f3*, a POU-domain transcription factor essential for hair cell maturation and survival [33], [34]. At E14.5, *in situ* hybridization of *Pou4f3* showed its expression in all vestibular sensory epithelia and the cochlea (Fig. 5k). The expression in the cochlea appeared to follow the expression pattern of *Atoh1*, starting from the base and progressing towards the apex. Interestingly, we found *Pou4f3* expression in control animals to be continuous in the inner hair cells but only in a few sporadic outer hair cells at this stage (Fig. 5k’). At E18.5, the expression became uniform in all hair cells in the control cochlea (Fig. 5m, m’). These data indicated that like *Atoh1* and *Barhl1*, the early expression of *Pou4f3* was differentially initiated in the two different types of hair cells, possibly related to the level and/or timing of *Atoh1* expression. In the CKO mutant ear, *Pou4f3* expression was reduced in all sensory epithelia with patchy loss (Fig. 5l, l’, n, n’), which was consistent with the loss of hair cells in the mutant.

Together, these data suggested that the expression of many genes related to hair cell development was differentially dependent on *Atoh1*. *Atoh1* directly or indirectly regulated essential transcription factors for normal hair cell development and maintenance, some of which apparently require sustained and more profound expression of *Atoh1*. Interestingly, even a transient expression of *Atoh1* may be sufficient for long-term expression of some structural genes such as *Myo7a* and *Myo6* (data not shown), which are also associated with loss of hair cells when mutated [35].

## Discussion

We present here the first genetically engineered animal model that directly affects both level and duration of *Atoh1* expression in the ear. Our data demonstrate that prolonged *Atoh1* expression at a yet to be defined level is needed to initiate proper differentiation of hair cells. We provide for the first time evidence that *Atoh1* regulates major aspects of hair cell development related to mechanosensory transduction. In addition, our data suggest that a certain level of *Atoh1* is needed for long-term maintenance of hair cells, in particular of inner hair cells, as limited *Atoh1* expression results in apparently non-functional hair cells that undergo progressive degeneration over time with an earlier and more complete loss of inner hair cells.

### *Atoh1-cre Atoh1* CKO mice provide a novel mouse model for studying hair cell regeneration

Animal models play a crucial role in biomedical research for investigating the mechanism of human diseases and developing treatments. Although the mouse has been the most frequently used animal model in many research areas, its use in hearing studies has been limited largely due to the high resistance of various experimental mouse strains to ototoxic drugs. Studies have shown notable differences in drug sensitivity between species [36], which precludes an effective, simple and reproducible approach using ototoxic drugs to eliminate hair cells in mice.

Previously, we use *Tg(Pax2-cre)* to conditionally delete *Atoh1* in the ear, which results in complete absence of differentiated hair cells in the organ of Corti [9]. These mice also show overall growth defects and can only survive up to about one month, which hinders the use of these mice for investigating hair cell regeneration at adult stages. Our current *Atoh1-cre Atoh1* CKO mice can live into adulthood without any gross phenotypes beyond deafness and some vestibular dysfunction. The patchy distribution of some partially differentiated hair cells with retention of significant spiral ganglion neurons that innervate those remaining cells more closely mimics the conditions in many human deafness patients, where hair cell loss is both progressive and less complete than in experimental models that chemically wipe out all hair cells simultaneously at a given time. Therefore, the mice characterized here for the first time provide an ideal system to establish conditions to transform the remaining partially differentiated cells into fully differentiated hair cells.

Loss of hair cells results in a featureless ‘flat epithelium’ within two weeks after birth in the *Pax2-cre Atoh1* CKO neonates. With a variable delay, this process also takes place in *Pou4f3* null mice [33], [34] or after chemically induced hair cell loss in neonates [37]. Our *Atoh1-cre Atoh1* CKO mice display similar ‘flat epithelium’ between the patches of hair cells. Our studies show loss of *Fgf10* expression in the gaps between hair cells and concomitant expanded expression of *Bmp4* in the region that presumably transforms to the ‘flat epithelium’ in later stages, suggesting that the properties of these cells shift from neuroepithelial to epithelial [38] and apparently depend on yet to be determined signals from the organ of Corti for continued expression. We propose that the alterations in the expression of these genes as well as possibly some other transcription factors crucial for the embryonic transformation of ectoderm into neurosensory development competent cells, such as *Pax2*, *Sox2*, *Eya1* and *Gata3* [39], [40], [41], [42], [43], [44], may underlie the lack of competence of these ‘flat epithelial cells’ to respond to Atoh1 protein to be transformed into hair cells [1]. Therefore, our mouse model may also be used to identify the transcription factors required for restoring the competence for neurosensory induction and ultimately to test at various stages if additional hair cells can form from the ‘flat epithelium’ between the patches. Since the hair cell loss is genetically mediated, the pattern of loss is highly reproducible, which would allow direct comparison between the treated and untreated ears to quantify the formation of additional hair cells.

### *Atoh1* plays a crucial role in regulating the expression of genes associated with hair cell mechanotransduction development

Our studies reveal profound defects in hair cell mechanotransduction development in *Atoh1-cre;Atoh1^f/f^* CKO ears, which had not been identified in previous work in *Atoh1* null or *Pax2-cre;Atoh1^f/f^* CKO due to the complete lack of hair cell differentiation in those mice. The progressive loss of stereocilia bearing hair cells may relate to continued cell death as well as deterioration of stereocilia with age. Previous work in *Pou4f3* mutants also show a progressive degeneration of initially near normal stereocilia over time and undifferentiated hair cells may persist for weeks in the cochlea and months in the vestibular organs [6]. In addition, even loss of cytoskeletal proteins such as Myo7a or Myo6 can result in loss of initially formed stereocilia followed by the loss of hair cells [35]. Such maturational changes in the seemingly rigid stereocilia are consistent with the emerging view of a highly dynamic molecular interaction for stereocilia maintenance [45], [46]. However, in comparison with these later mutants, the *Atoh1-cre;Atoh1^f/f^* CKO cochlea shows a more severe disorganization of developing stereocilia and an earlier onset of stereocilia degeneration. These evident differences suggest that *Atoh1* directly or indirectly regulates most aspects of stereocilia development and maintenance. Further comparative genomic analysis of the *Atoh1-cre;Atoh1^f/f^* CKO mutants will help to identify the immediate downstream genes responsible for the multiple functions of *Atoh1* in regulating hair cell differentiation and to determine how these genes relate to those identified in the developing cerebellum [47].

### The level and duration of *Atoh1* expression may play a role in the hair cell type specific differentiation and viability

The function of the organ of Corti critically depends on the well-ordered distribution of two hair cell types. Inner hair cells, situated medial to the pillar cells, are the sole mechanoelectric transducers that convert local hydrodynamic events elicited by sound into resting potential changes to be transmitted by type I spiral ganglion cells to the cochlear nuclei. In contrast, the more abundant outer hair cells are responsible for the localized signal amplification for enhanced sensitivity of hearing [48]. These two different types of hair cells are surrounded by seven different types of supporting cells and form a highly organized structure that is functionally important. Hence, full restoration of hearing requires not only regenerating hair cells but also re-establishing this orderly cellular distribution, for which the molecular basis of differentiation is essentially unknown.

**Figure 6:**
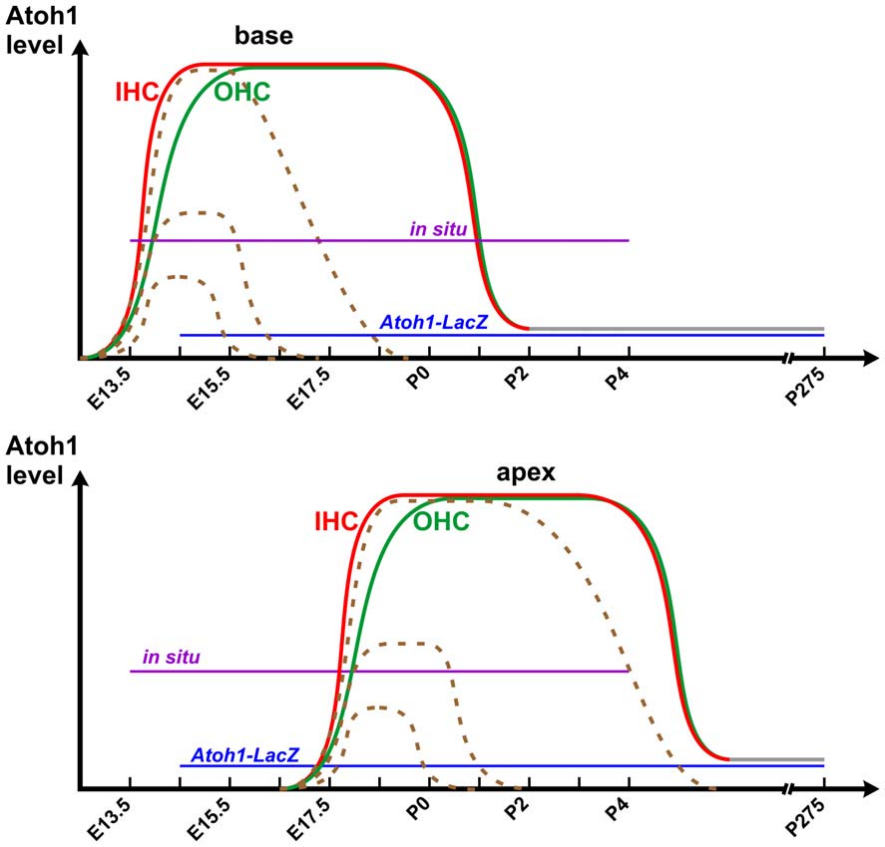
A working hypothesis shows the differential expression of *Atoh1* in inner hair cells (IHC) and outer hair cells (OHC). Based on our *in situ* hybridization data, *Atoh1* is expressed earlier and at a higher level in IHCs (red line) than OHCs (green line), first in the base (top) followed by the apex (bottom). At early postnatal stages, *Atoh1* is downregulated to below the detection threshold of *in situ* hybridization, but a low level of *Atoh1* expression (grey lines) likely is sustained and can be detected by *Atoh1-LacZ* as late as P275 [14]. In the CKO mutant cochlea, the *Atoh1* expression is reduced to a range of different levels shortly after its upregulation and is then eliminated after a few days (dashed lines). Some cells at the apex express *Atoh1* at a level close to control hair cells and can survive to adult stage.

While some correlation with differential gene expression such as *Fgf8* in inner hair cells is well known [26], [49], no causality has been proved for any gene expression that is directly linked to the development of the different types of hair cells. Using the *Atoh1* enhancer to drive the expression of GFP, it has been suggested that onset of *Atoh1* expression correlates with the development of different types of hair cells [50], [51]. However, this reporter shows delayed *Atoh1* expression compared to *in situ* hybridization or *Atoh1-LacZ* and thus remained inconclusive. We show here by *in situ* hybridization in both whole mount and sections that *Atoh1* mRNA is expressed earlier and at a higher level in the inner hair cells (Fig. 5a–b’, 6). This is consistent with the data obtained using *Atoh1-lacZ* [32]. Both detection approaches may truly reflect the differential upregulation of *Atoh1* in the different types of hair cells, although the detection threshold may vary (Fig. 6). We also show that limited expression of *Atoh1* as obtained with our CKO mouse line results in rapid and near complete loss of all inner hair cells while many outer hair cells are retained for days and weeks (Fig. 4).

We recently showed that deletion of another bHLH gene, *Neurod1,* results in transformation of some outer hair cells in the apex into inner hair cell-like cells, including the expression of *Fgf8* in these cells [30]. This transformation correlates with a premature upregulation of *Atoh1* expression in the organ of Corti, suggesting that *Neurod1* suppresses *Atoh1* expression in the ear, comparable to the cerebellum [52]. Based on these previous gain-of-function data and the current loss-of-function data, we suggest that the level and/or onset of *Atoh1* expression may play a role in hair cell type specification. The molecular mechanism requires further study.

Our data on vestibular hair cell development show an even wider range of stereocilia morphology, suggesting that variability in the delay of recombination and initial *Atoh1* expression is even greater than in the organ of Corti hair cells. We have not yet established whether the early loss is also particularly prominent in one of the two types of vestibular hair cells as we could not show expression of the few proteins used in the past to distinguish between type I and II vestibular hair cells [53] There is also an unexpected gradient of hair cell loss in various vestibular sensory epithelia with the saccule being more susceptible than any other vestibular epithelium. We assume that the interplay between cell cycle exit, initial expression of *Atoh1* and the activation of the *Atoh1-cre* construct will determine how much *Atoh1* mRNA will be generated before the floxed genes are recombined. Clearly, a detailed assessment of levels of *Atoh1* expression at the cellular level is needed to verify this assumption.

In summary, susceptibility of hair cells to low levels and early loss of *Atoh1* follows a simple sequence in the organ of Corti: IHC>>OHC>>flat epithelium. Our data suggest that some of the recurring difficulties in various studies using *Atoh1* to generate hair cells may relate to uncontrollable fluctuations of either level, or duration of *Atoh1* expression, or both. Generating new mouse lines using inducible *cre* could provide mouse models with various *Atoh1* expressions, which are needed to further define the level and duration of *Atoh1* for successful regeneration of viable hair cells.

## Supporting Information

Figure S1 **Hair cells are reduced in** ***Atoh1*** **CKO vestibular sensory epithelia, in particular the saccule. (a–d’)** Myo7a immunohistochemistry (red) shows hair cells in E14.5 utricle and saccule. Compared with control ears, the CKO mutant ears have fewer Myo7a-positive cells, particularly in the saccule (d). Higher magnification images also show disorganization of the hair cells in the mutant (b’, d’). **(e–f’)** At E18.5, the reduction of Myo7a-positive cells is more obvious in almost all the vestibular endorgans except the utricle. The nerve fibers shown by Tubulin immunolabeling (green) innervate the remaining hair cells. AC, anterior crista; HC, horizontal crista; PC, posterior crista; S, saccule; U, utricle. Bar indicates 100 µm in a, b, c, d, e-f’ and 10 µm in a’, b’, c’ d’.
(TIF)

Figure S2 **Loss of** ***Atoh1*** **results in abnormal stereocilia development in vestibular hair cells. (a-f)** Espin immunolabeling (red) shows hair cell stereocilia in vestibular sensory epithelia of control and CKO mutant neonate mice. All five epithelia in the mutant have Espin-positive cells (b), but the number of cells is prominently reduced compare with control ear (a). The stereocilia in mutant posterior crista hair cells appear longer (compare b’ with a’, and e with c). The Espin staining is vastly reduced in the mutant saccule (compare d with f), due to reduction in both the number of cells with stereocilia and the number of stereocilia in each cell. The Tubulin antibodies also label the kinocilia in the hair cells at these stages (green), which can be found without associated stereocilia in the mutant ears (arrows in f). **(g–i’”)** SEM images show abnormal stereocilia development in the mutant vestibular endorgans. In P7 mutant saccule, the cells with stereocilia-like structures are reduced (h) and the number of stereocilia in each cell is also reduced (h’, h”). In the posterior crista of a P327 mutant ear, the few remaining hair cells have very long stereocilia and kinocilia (i’–i’”, arrows in i”). AC, anterior crista; HC, horizontal crista; PC, posterior crista; S, saccule; U, utricle. Bar indicates 100 µm in a, b, 10 µm in a’, b’, c-i”, and 1 µm in i’”.
(TIF)

Figure S3 ***Atoh1*** **CKO mice are deaf at 3 months of age.** Representative ABR (auditory brainstem response) recordings of control mice display normal patterns induced by click stimuli at five different sound pressure levels (**a**). When induced by same stimuli, the CKO mutant mice show complete lack of response (**b**).
(TIF)
